# Supplementary material for: Organization and Characterization of the Promoter Elements of the rRNA Operons in the Slow-Growing Pathogen Mycobacterium kumamotonense
Source: Genes (Basel). 2023 Apr 30;14(5):1023. doi: 10.3390/genes14051023 (PMC10218544; doi:10.3390/genes14051023)
Supplement: Supplementary file 1 [file genes-14-01023-s001.zip › genes-2334730-supplementary/Supplementary material/Table S1.pdf]

Table S1. Primer sequences

Primer	Sequence (5´- 3´)	Tm (°C)	Reference
RAC1	TCGATGATCACCGAGAACGTGTTC	55	[10]
RAC8	CACTGGTGCCTCCCGTAGG	55	[25]
TYRS3	TGCAGGCAACGACTACGT	55	This work
cR103	CGACTTGCATGTGTTAAGC	55	[22]
JY15	CACACTATTGAGTTCTC	55	[22]
rrs-Fw	TTGGGTTAAGTCCCGCAACGA	60	This work
rrs-Rv	GCCCTGGACATAAGGGGCAT		This work
P1-Fw	GCCGAGCAGTCTGGAAG	60	This work
P1-Rv	CGGAAGCCGGAGTTGAG		This work
P2-Fw	CGTAACTTAGTGCACGTC	60	This work
P2-Rv	GTGCGGACAAGTGAAAC		This work
ahpC-Fw	ACGAGATCCAGTTCGTGT	60	This work
ahpC-Rv	TCGCCCTTCTTCCAGTT		This work
groEL-Fw	TTCCTGTCGGCGTACTT	60	This work
groEL-Rv	ACCGGGACAAGATCAGTT		This work
