# Supplementary material for: Organization and Characterization of the Promoter Elements of the rRNA Operons in the Slow-Growing Pathogen Mycobacterium kumamotonense
Source: Genes (Basel). 2023 Apr 30;14(5):1023. doi: 10.3390/genes14051023 (PMC10218544; doi:10.3390/genes14051023)
Supplement: Supplementary file 1 [file genes-14-01023-s001.zip › genes-2334730-supplementary/Supplementary material/Table S2.pdf]

Table S2. Consensus promoter sequences of *M. kumamotonense* and *M. tuberculosis* sigma factors

Consensus sequences	Promoter sequence		References
	**-35 sequence**	**-10 sequence**	
M. kumamotonense P1 *rrnB*	TTGACT	TAACTT	This work
M. tuberculosis SigA (σ^A) binding site	TTGACT	TATACT	[31]
M. kumamotonense PCL1 *rrnA*	GTAGCC	TCAACT	This work
M. tuberculosis SigD (σ^D) binding site	GTACCGCT	CGTTAT	[32]
