# Supplementary figures and images for: Organization and Characterization of the Promoter Elements of the rRNA Operons in the Slow-Growing Pathogen Mycobacterium kumamotonense
Source: Genes (Basel). 2023 Apr 30;14(5):1023. doi: 10.3390/genes14051023 (PMC10218544; doi:10.3390/genes14051023)

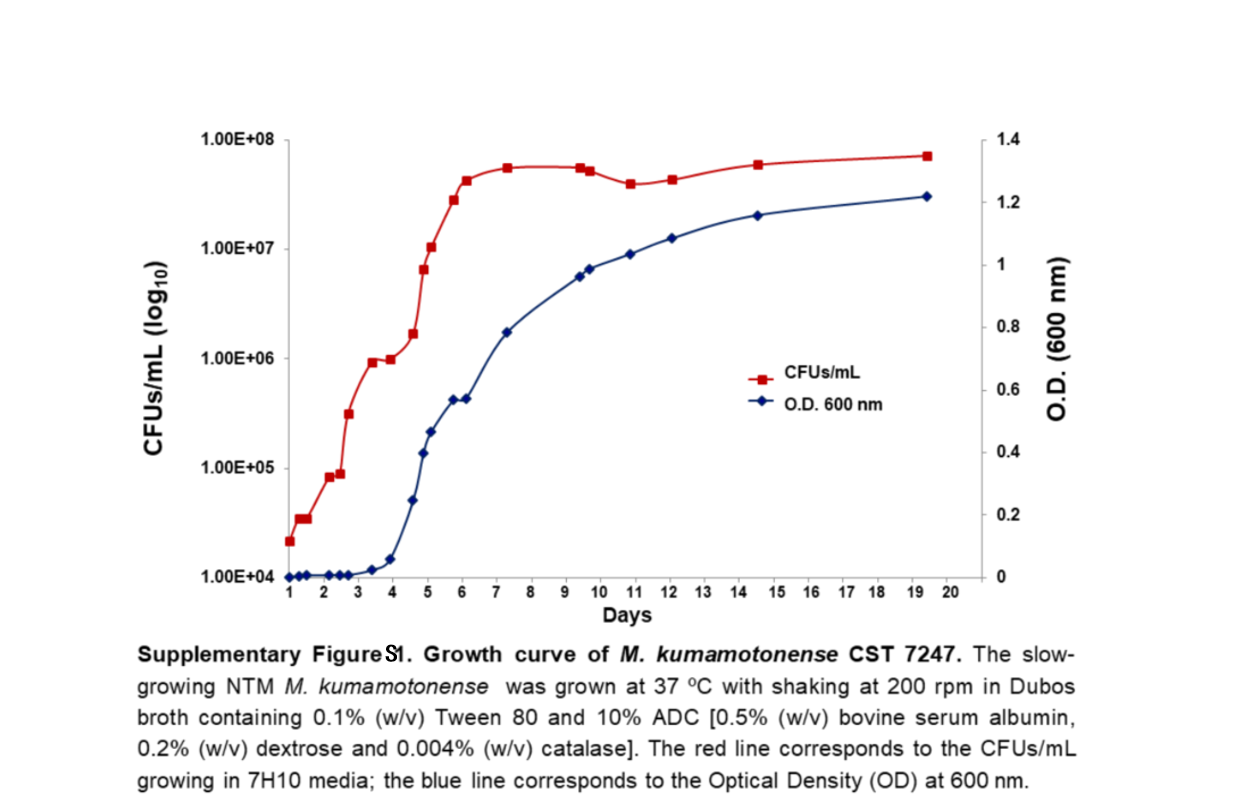

Supplement: Supplementary file 1 [file genes-14-01023-s001.zip › genes-2334730-supplementary/Supplementary material/Figure_S1.png]

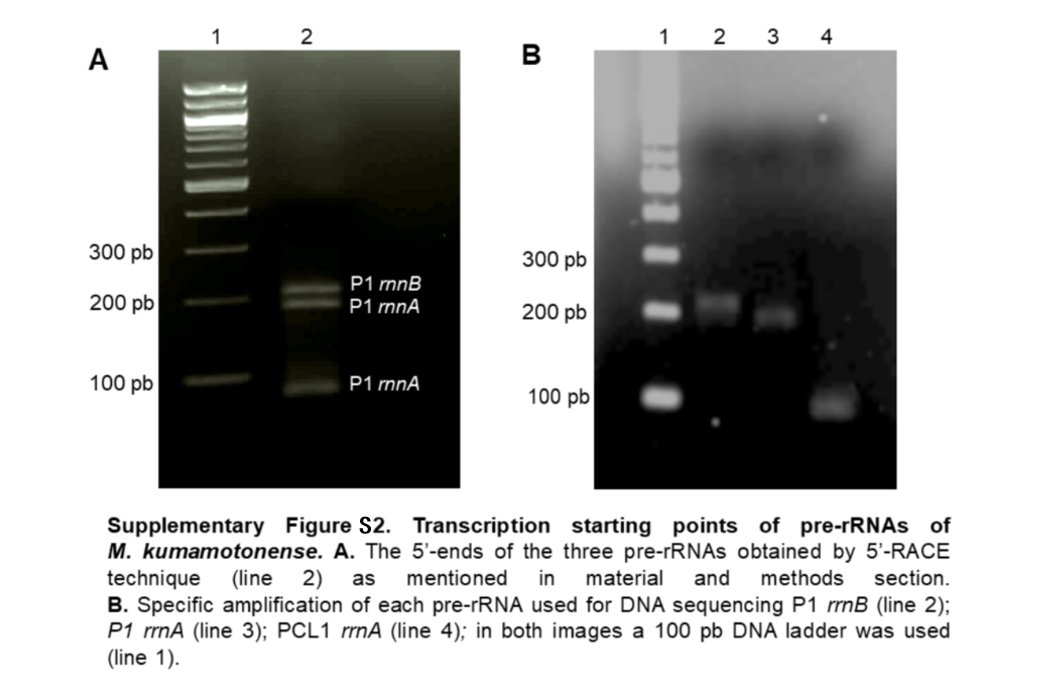

Supplement: Supplementary file 1 [file genes-14-01023-s001.zip › genes-2334730-supplementary/Supplementary material/Figure_S2.png]

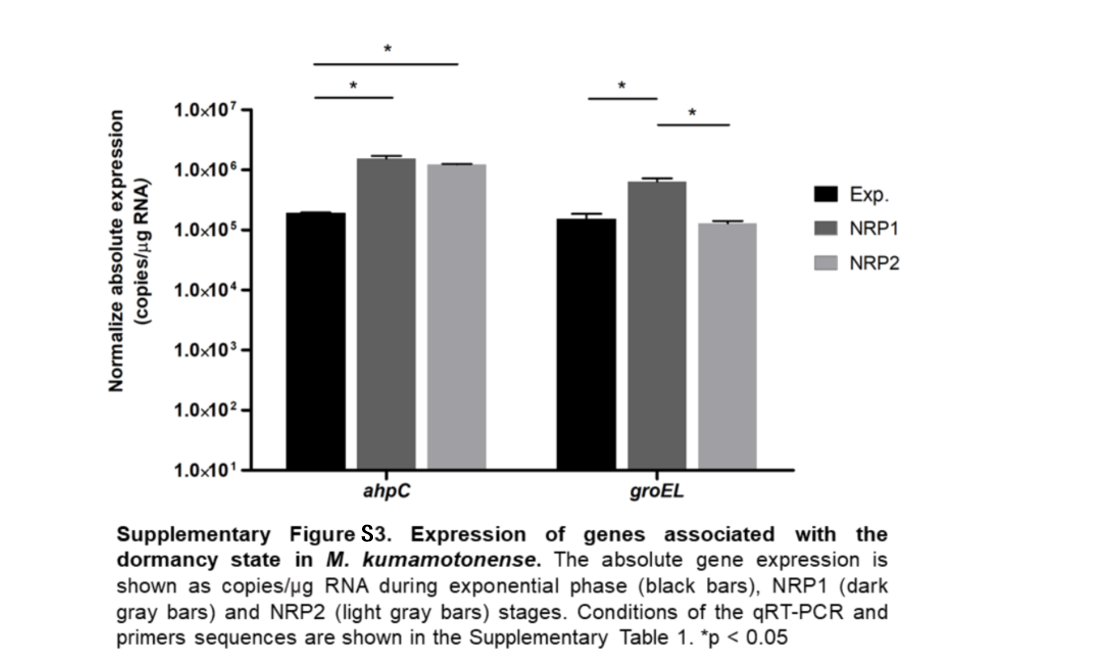

Supplement: Supplementary file 1 [file genes-14-01023-s001.zip › genes-2334730-supplementary/Supplementary material/Figure_S3.png]
